# Protease Inhibitors from Marine Actinobacteria as a Potential Source for Antimalarial Compound

**DOI:** 10.1371/journal.pone.0090972

**Published:** 2014-03-11

**Authors:** L. Karthik, Gaurav Kumar, Tarun Keswani, Arindam Bhattacharyya, S. Sarath Chandar, K. V. Bhaskara Rao

**Affiliations:** 1 Environmental Biotechnology Division, School of Bio Sciences and Technology, VIT University, Vellore, Tamil nadu, India; 2 Immunology Lab, Department of Zoology, University of Calcutta, Kolkata, West Bengal, India; 3 Genetics Division, School of Bio Sciences and Technology, VIT University, Vellore, Tamil nadu, India; Institut de Pharmacologie et de Biologie Structurale, France

## Abstract

The study was planned to screen the marine actinobacterial extract for the protease inhibitor activity and its anti- Pf activity under *in vitro* and *in vivo* conditions. Out of 100 isolates, only 3 isolates exhibited moderate to high protease inhibitor activities on trypsin, chymotrypsin and proteinase K. Based on protease inhibitor activity 3 isolates were chosen for further studies. The potential isolate was characterized by polyphasic approach and identified as *Streptomyces* sp LK3 (JF710608). The lead compound was identified as peptide from *Streptomyces* sp LK3. The double-reciprocal plot displayed inhibition mode is non-competitive and it confirms the irreversible nature of protease inhibitor. The peptide from *Streptomyces* sp LK3 extract showed significant anti plasmodial activity (IC_50_: 25.78 µg/ml). In *in vivo* model, the highest level of parasitemia suppression (≈45%) was observed in 600 mg/kg of the peptide. These analyses revealed no significant changes were observed in the spleen and liver tissue during 8 dpi. The results confirmed up-regulation of TGF-β and down regulation of TNF-α in tissue and serum level in *PbA* infected peptide treated mice compared to *PbA* infection. The results obtained infer that the peptide possesses anti- Pf activity activity. It suggests that the extracts have novel metabolites and could be considered as a potential source for drug development.

## Introduction

Malaria is a highly infectious disease caused by a protozoan parasite of the genus Plasmodium. These parasites are transmitted by the bite of infectious female *Anopheles* sp mosquitoes. There are totally five species of Plasmodium associated with malarial fever viz., *P. falciparum*, *P. vivax*, *P. ovale*, *P. malariae* and *P. knowlesi*. Among them, *P. falciparum* is highly virulent and it is the predominant agent in Africa. While, *P. vivax* is comparatively less virulent and is more prevalent throughout the world and remaining three species are associated with the minor outbreaks in several parts of the world. Malaria is a major cause of morbidity and mortality and it is projected that around 3.3 billion people were at risk of malaria in 2010. Likewise, among 91% of deaths are estimated in the WHO African Region, with children under five years of age and pregnant women being severely affected [1]. World Malaria Report (2012) summarizes that 106 countries are malaria-endemic in 2011 [2].

Three different approaches were considered for the control of more virulent malarial parasite, *Plasmodium falciparum*. They are widely exploited development of effective vaccines, vector control and development of new drugs. It is very difficult to develop a vaccine due to their exhibition of their multiple antigenicity. Based on several factors, the vector control shows limited success. On the other hand, there is an increasing resistance of malarial parasites to the existing drug hence, there is an urgent need demand for new antimalarial agents [3], [4].

Due to the evolution of drug resistance in *Plasmodium* sp, which necessitates the need for new drugs, ideally directed against new targets such as heme and malarial proteases. The life cycle of malarial parasite exhibits two stages: exoerythrocytic cycle and erythrocytes life cycle. The erythrocytes life cycle was responsible for all clinical manifestations and it begins when free merozoites invade erythrocytes. The free merozoites will enter into the RBC cells and develop from small ring-stage organisms to larger, more metabolically active trophozoites followed by multinucleated schizonts [5]. The schizonts will ruptures the erythrocytes and releases 30,000 invasive merozoites in *P. falciparum*, 10,000 for *P. vivax* and *P. ovale* and 2,000 for *P. malariae.* This step is called as egress. At this stage, proteases are required for the rupture and subsequent invasion of erythrocytes by merozoite stage parasites and for the degradation of hemoglobin by intraerythrocytic trophozoites.

The merozoites form of *P. falciparum* express a number of merozoite surface proteins (MSPs). These may be considered as target antigens for vaccine preparation [6]. The merozoites synthesize a B195-kDa glycosyl phosphatidy- linositol-anchored precursor that assembles as a complex with two peripheral membrane proteins such as MSP6 and MSP7 [7]–[10]. This complex (MSP1/6/7) is uniformly present in the merozoite surface and it initiates the erythrocyte invasion [11]. This complex was involving ‘primary’ proteolytic cleavage events earlier to egress stage [12] and the cleavage products remain associated with the surface of the released merozoite, to the complex is finally shed at the point of erythrocyte invasion in an essential secondary processing step by the action of a membrane-bound parasite protease called PfSUB2 [13]. The primary proteolysis and the positional conservation of the cleavage sites in MSP1 orthologues across the *Plasmodium* genus [14] proposed that prime processing is essential for the function of the MSP1/6/7 complex and for merozoite viability.

The exonemes, specialized merozoite organelles releases the subtilisin-like serine protease called PfSUB1 [15] and it mediates the proteolytic maturation of members of a family of abundant, papain-like putative proteases called SERA, previously implicated in egress [16]. The inhibition of PfSUB1 prevents SERA maturation and block egress. This indicates a role for PfSUB1 in triggering egress, probably through activation of the SERA enzymes.

Enzyme inhibitors are the third important product of marine actinobacteria. So far, it is used for the study of enzyme structures and reaction mechanisms, but recently it has been used in pharmacology [17]. These selective inhibitors can be used as a powerful tool for inactivating target proteases in the pathogenic processes of human diseases such as malaria, emphysema, arthritis, pancreatitis, thrombosis, high blood pressure, muscular dystrophy, cancer, and AIDS [18]. Enzyme inhibitors from marine microorganisms were sparsely studied. However, terrestrial isolated *Streptomyces* is a one of the potential producers of enzyme inhibitors [19]. The isolation of novel enzyme inhibitor from terrestrial sources is rare hence marine actinobacteria will provide new potential inhibitors.

Proteases are essential constituents found in prokaryotes, fungi, plants and animals. Serine, cysteine, metalloproteases is widely spread in many pathogenic parasites, where they play critical functions related to evasion of host immune defenses, acquisition of nutrient for growth and proliferation, facilitation of dissemination or tissue damage during infection [20]. Thus, proteases play a foremost role in pathogenesis. Moreover, protease enzymes are used for a long time in various forms of clinical therapies. Their application in medicine is gaining more and more attention as several clinical studies are indicating their benefits in oncology, inflammatory conditions, blood rheology control and immune regulation. Hence, this study was focused on the screening of the protease inhibitor from marine actinobacteria for anti- Pf activity under *in vitro* and *in vivo* conditions.

## Materials and Methods

### Ethics statement

Animal experiments were carried out as per the guidelines of the Committee for the Purpose of Control and Supervision of Experimental Animals (CPCSEA), Government of India (Registration No: 885/ac/05/ CPCSEA) and as approved by the Institutional Animal Ethics Committee (IAEC) University of Calcutta, and confirms with the Guide for the Care and Use of Laboratory Animals published by the US National Institutes of Health (NIH Publication No. 85–23, revised 1985).

### Media, chemicals and reagents

All the media were purchased from Himedia, India. Giemsa's azur eosin methylene blue solution and Hematoxylin and eosin stains for microscopy were obtained from Merck KGaA Frankfurter Str., Germany. A phosphate buffer solution (PBS), sodium bicarbonate, sodium azide, RNase A, NBT (nitro-blue tetrazolium chloride) and BCIP (5-bromo-4-chloro-3′-indolyphosphate p-toluidine salt) (Cat# RM 578, RM 2577) were procured from Himedia chemicals, India. Antibodies against TGF-β, TNF-α, β-actin, AP-linked anti-rabbit, AP-linked anti-mouse secondary antibodies were purchased from the Cell signaling Technology Cell Signaling Technology, Inc (Danvers, MA, USA). Pre stain molecular weight protein markers were purchased from Bangalore Genei (India) and the remaining chemicals were purchased in an analytical grade of the highest purity (India). All solutions were prepared with commercial reagents of at least pro-analysis quality and with sterilized 18 MΩ milliQ water. When necessary, the specific origins of reagents are listed in the text.

### *In silico* molecular docking analysis

To predict the inhibiting characteristics of respective inhibitors/ligands (*S. albogriseolus*, *S. longisporus*, *S. fradiae*, *S. lividans* and *S. griseus*) on PLASMEPSIN- II and FALCIPAIN-2 receptors, *in silico* molecular docking approach was opted. Initially before docking calculations, both receptors and ligands were subjected to energy minimization by GROMOS97 available in a Swiss PDB viewer. For the above *in silico* approach, Hex Server (http://hexserver.loria.fr), an interactive molecular graphics program was used for calculating and visualizing feasible docking modes of pairs of macromolecules. It adopts Spherical Polar Fourier (SPF) correlations to increase speed of the docking calculations. In the first step as an input, respective PDB files of ligands and receptors were specified in the Hex server [21]. Further, the docking correlation type calculation parameters were set to “Shape and electrostatics” mode. Later, the origin and interface residues for the docking calculations were set to their respective default values. Further, the range angles for receptor and ligands were set to 180°. The step sizes for the docking calculations were set to 10. Finally, the docking evaluation solutions were set to evaluate 10 best docking solutions. All the docking calculations were performed using GPU mode. The best docking orientations were selected based on the binding energy (kJ/mol) for the further intermolecular interaction studies. The results were analyzed with Hex 6.0, a stand-alone graphical application to visualize the intermolecular interactions of receptors and ligands.

### Actinobacteria strains

Hundred actinobacteria strains were isolated from the island of Nicobar (9° 09′ N, 92° 49′ E). These isolation procedures were already published [22]. Further, these isolated strains were used for screening to detect the protease inhibitor production. These strains were designated as LK1-LK100. All the strains were inoculated into 50 ml SS medium in 250 ml Erlenmeyer flasks containing the sea water 50%, distilled water 50%, pH 7.5 and incubated for 2 days in a rotary shaker incubator (200 rpm) at 28°C. The seed inoculums (10%) were transferred into 200 ml production medium in 1 L Erlenmeyer flasks. The inoculated cultures in the production medium were incubated for 72 h on a rotary shaker (2000 rpm) at 28°C. After fermentation the broth was centrifuged at 4000 rpm for 10 min and the filtrate was separated.

### Assay for protease inhibitor activity

To a mixture of mercuric chloride, phosphate buffer and trypsin solution, marine actinobacterial extracts were added. After adjusting the pH to 7.5 BAPNA (N-α-benzoyl-DL-arginine-p-nitroanilide) was added and incubated at 37°C for 20 mins. After incubation, 5% of the TCA was added and incubated at room temperature for 20 mins. Later, the solution was filtered with Whatmann no. 1 filter paper and the absorbance was taken at 280 nm. The inhibiting activity of protease inhibitor was expressed using following formula.

Inhibiting activity (%) =  C-T/C X 100

### Kinetics of protease inhibition by protease inhibitor

The mode of inhibition of protease inhibitor against trypsin activity was measured with increasing concentrations of BAPNA (0.5,1,2 and 4 mM) as a substrate in the absence and presence of protease inhibitors at 0.5 mg/ml and 1 mg/ml. Optimal amounts of protease inhibitor were determined based on the enzyme inhibitory activity assay. Mode of inhibition of protease inhibitor was determined by Lineweaver–Burk plot analysis of the data calculated following Michaelis-Menten kinetics.

### Dialysis for reversibility of protease inhibitor action

Trypsin (100 U/ml) was incubated with protease inhibitor (23.5 mg/ml) in 0.5 ml of tris HCL buffer (10 mM, pH 7.5) for 2 h at 37°C and dialyzed against tris HCL buffer (1 mM, pH 7.5) at 4°C for 24 h, changing the buffer every 12 h. Another premixed-enzyme solution (0.5 ml) was kept at 4°C for 24 h without dialysis for the control experiment. Reversibility of protease inhibitor has been determined by comparing the residual enzyme activity after dialysis with that of non-dialyzed one.

### Identification of the actinobacterial Isolate

The strain was screened based on the protease inhibitor production and the efficient isolate was subjected to molecular characterization based on 16s rRNA sequencing chromous biotech, Bangalore, India. The 16s rRNA fragment was amplified using PCR polymerase. The PCR product was sequenced bi-directionally using the forward (5′-AGAGTRTGATCMTYGCTWAC-3′) and reverse (5′-CGYTAMCTTWTTACGRCT-3′) primers. The sequence was analysed by ABI3730XL capillary DNA sequencer (ABI Prism 310 Genetic Analyzer, Tokyo, Japan). Further, the genus and species were successfully identified.

### Extraction, purification and characterization of protease inhibitor

The *Streptomyces* sp LK3 was subjected to fermentation for 7 days in production medium. After fermentation, the supernatant was collected and purified by four sub-sequential steps viz., ammonium sulphate precipitation, dialysis, Ion exchange chromatography and preparative HPLC chromatography. The functional group of the compound was measured using Fourier transform infrared (FT-IR) spectroscopy (Thermo Nicolet -330, USA) using a KBr pellet. The structure of the compound was established by using the spectral data obtained from gas chromatography (Perkin Elmer, Claeus 680) -mass spectrometry (Claeus 600) at a flow rate of 1 ml/min with a carrier gas of helium and Liquid Chromatography-Mass Spectrometer (LC-MS) (Thermo LCQ Deca XP MAX). The proton NMR (^1^H NMR), carbon NMR, H,H-COSY and HSQC (^13^C NMR, V Bruker Avance III 500 MHz (AV 500)) spectra of the compounds were obtained by using a dimethyl sulfoxide d6 (DMSO-d6) as solvent.

### *In vitro* anti- Pf activity

*P. falciparum* isolates NZR-2 was obtained from National Institute of Malaria Research, New Delhi, India. The experiment was carried out in a 96-well microtitre plate by adding 150 µl of infected human red blood cell suspension to each well. The peptide was dissolved separately in RPMI-1640 to obtain a concentration range of 20, 40, 60, 80 and 100 µg/ml and 50 µl of each dilution was poured into individual wells separately. Artemisinin was used as a positive control and water as a negative control. The microtitre plate was incubated for 48 hours. The percentage of parasitemia was determined microscopically after Giemsa staining.

### *In vivo* anti- Pf activity

Male Swiss albino mice (∼25 g each; aged 6 to 8 weeks) were maintained in sterilized cages and absorbent media; food and water were provided ad libitum. Animal experiments were carried out as per the guidelines of the Committee for the Purpose of Control and Supervision of Experimental Animals (CPCSEA), Government of India (Registration No: 885/ac/05/ CPCSEA) and as approved by the Institutional Animal Ethics Committee (IAEC) University of Calcutta, and confirms with the Guide for the Care and Use of Laboratory Animals published by the US National Institutes of Health (NIH Publication No. 85–23, revised 1985).

Parasite strain *Plasmodim berghei* ANKA obtained from National Institute Malaria Research Center, New Delhi. Parasitized mouse red blood cells (pRBC) from a liquid N_2_ preserved stabilize were injected (1×10^6^ pRBC, in 100 µl phosphate buffer solution) into mice of the same background. Mice were infected with *PbA* (1×10^6^ pRBC), in 100 µl PBS by intraperitoneal injection, after amplification an equal number of uninfected erythrocytes were injected into control mice. Parasitemia was monitored daily in all experimental groups by Giemsa-stained thin blood smears made from tail snips. Survivability and parasitemia of mice (n = 60) were also observed daily. The percentage of parasitemia was calculated as follows: Parasitemia (%)  =  [(number of infected erythrocytes)/(total number of erythrocytes counted)] x 100.

### Experimental groups parasite clearance and curative efficacy

For optimization of drug concentration, a stock solution of LK1, LK2 and LK3 (5000 mg/ml) were dissolved in (PBS), and 50 mice received 100 µl i.p. injections each of LK1, LK2 and LK3 (600, 800, 1000 and 1200 mg/kg/ BW respectively) from 2 dpi till the last day of survival. The control group received injections of vehicle on the same treatment schedule. Parasitological evaluation, survivability indicated that LK1 (800 mg/kg/BW), LK2 (1000 mg/kg/BW) and LK3 (600 mg/kg/BW) was the best drug concentration. Parasitamia and survivability in mice were monitored daily (n = 60). The experimental animals were divided into groups such as (1) uninfected control, (2) *PbA* (1×10^6^ pRBC) infected, *PbA* infected and treated with LK1 (800 mg/kg/BW) (3) *PbA* infected and treated with LK2 (1000 mg/kg/BW) and (4) *PbA* infected and treated with LK3 (600 mg/kg/BW). For an analysis on respective dpi, 5 mice from each experimental group were used.

The ‘8-day suppressive test’ was adopted for the determination of the parasite clearance and curative efficacy, respectively. The doses for LK1 (800 mg/kg/BW) (3) *PbA* infected and treated with LK2 (1000 mg/kg/BW) and (4) *PbA* infected and treated with LK3 (600 mg/kg/BW). Groups of 5 mice each were administered vehicle only. Tests were performed in a 8 day Suppressive standard test. The *Plasmodium berghei* is most widely employed in the rodent malaria parasite.

Thin smears of blood films were obtained from the peripheral blood on the tail from each mouse on day four after infection [23], [24]. The smears were placed on microscope slides, fixed with methanol and stained with Gemsa at pH 7.2, for parasitemia. The microscope had an Ehrlich's eyepiece and a nose diaphragm showing about 100 red blood cells per field. The number of parasitized erythrocytes in each of the 10–50 such fields were counted three times and the average was calculated to give the Parasitemia of each individual animal. Percentage of suppression was calculated by using the following formula [23], [24], [25]. 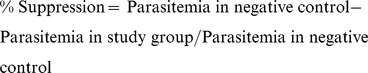



### Histopathology of spleen

The spleen was aseptically removed from each host and immediately washed in phosphate buffered saline (PBS, pH 7.4). The tissues were then fixed for 24 h in buffered formaldehyde solution (10% in PBS) at room temperature, dehydrated by graded ethanol, and embedded in paraffin (MERCK, solidification point 60–62°C). Tissue sections (5-µm thickness) were then deparaffinized with xylene, re-hydrated with graded alcohols (100–50% ethanol), stained with eosin and hematoxylin, and then mounted in DPX resin (Merck, Mumbai, India). Digital images were captured with Olympus CAMEDIA digital camera, Model C-7070 wide zoom.

### Western blot analysis and cytokine assays

The mechanisms were elucidated by Western blot analysis and cytokine assays as described earlier [26].

### Statistical analysis

Values between groups on same or different dpi were analyzed using one or two way ANOVA. All values are shown as mean ± SD, except where otherwise indicated. Data were analyzed and when appropriate, the significance of the differences between mean values was determined using Student's test. *P values <0.05 were considered significant for all statistical analysis, otherwise stated.

## Results and Discussion

### Estimation of protease inhibitors efficacies using *in silico* molecular docking

**Figure 1:**
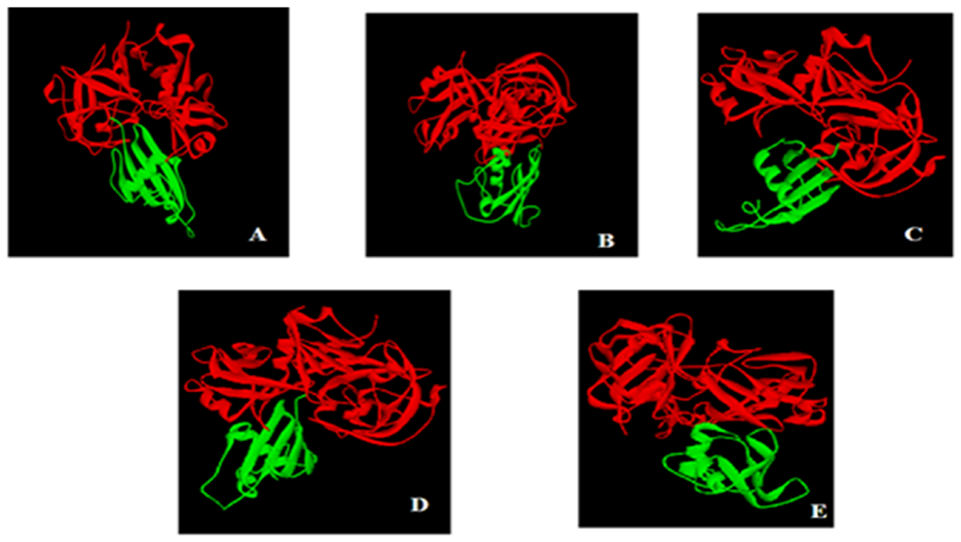
Intermolecular interactions between PLASMEPSIN- II and Protease inhibitor of marine actinobacteria A. *S. albogriseolus* PI; B. *S. longisporus* PI; C. *S. fradiae* PI; D. *S. lividans*; E. *S. griseus.* Enzyme PLASMEPSIN-II (indicated in red color) and their corresponding inhibitors (indicated in green color) are represented in ribbon configuration.

To validate the efficacy of protease inhibitors from marine actinobacteria as a suitable targets for the malarial protease, five inhibitors (*S. albogriseolus*, *S. longisporus*, *S. fradiae*, S. *lividans* and *S. griseus*) were assayed against PLASMEPSIN- II and FALCIPAIN-2 using Hex server (Table.1). Docking calculations revealed that *S. albogriseolus* have a probable tendency to inhibit the PLASMEPSIN- II receptor effectively and may outperform the other inhibitors. On the other hand, *S. longisporous* tends to inhibit FALCIPAIN-2 receptors, when compared to the other inhibitors. The preliminary study revealed that the protease inhibitors from five actinobacteria inhibit the respective receptors more effectively (Fig. 1). Hence, we had hypothesized the inhibiting characteristics of protease inhibitors obtained from marine actinobacteria comparatively and their roles in malaria chemotherapeutic development were elucidated using *in silico* studies.

**Table 1:** *In silico* studies of protease inhibitor from marine actinobacteria.

Receptors	Ligands-Binding Affinity(kJ/mol)				
	*A.S. albogriseolus*	*B.S. longisporus*	*C.S. fradiae*	*D.S. lividans*	*E.S. griseus*
**PLASMEPSIN- II**	−692.4540	−458.0594	−440.7606	−574.2310	−673.7805
**FALCIPAIN-2**	−192.4911	−590.0973	−269.6229	−420.6248	−491.3755

The docking revealed the *S. albogriseolus* has a tendency to inhibit the PLASMEPSIN- II receptor effectively. In addition, *S. longisporous* tends to inhibit FALCIPAIN-2 receptors.

### Screening of protease inhibitor producing marine actinobacteria

**Figure 2:**
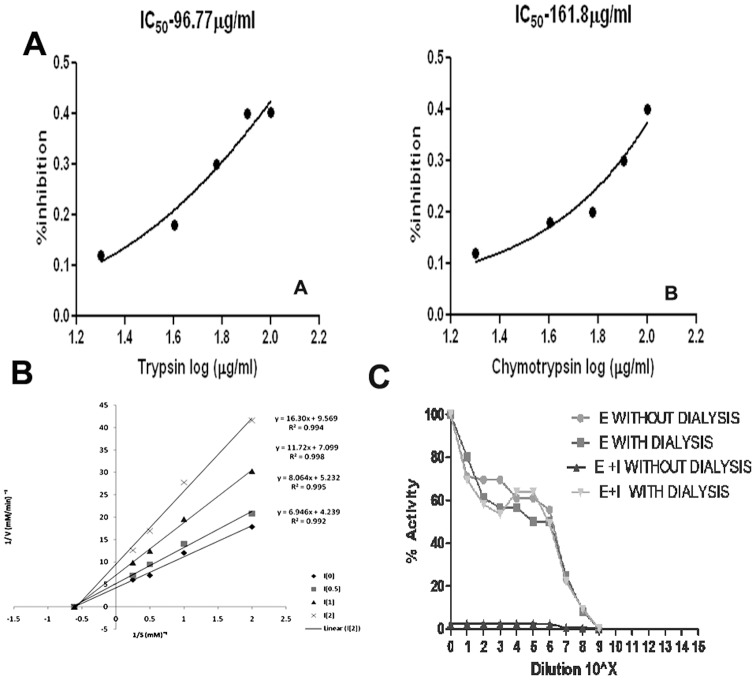
*Streptomyces* sp LK3 strain showed high protease inhibitor activity against trypsin and chymotrypsin with IC_50_ values of LK3 strain A. trypsin (96.77 µg/ml) and B. chymotrypsin (161.8 µg/ml) B. Mode of protease inhibition by peptide. The double-reciprocal plot displayed non-competitive inhibition mode C. Reversibility of peptide action was confirmed by the continuous steep decline in Vmax with an increase in inhibitor concentration with Km value remaining confined.

Among the 100 isolates tested for protease inhibitor activity, LK-1, LK-2 and LK-3 strain showed substantial protease inhibitor activity. The optimized 3 strains were subjected to fermentation and the protease inhibitor extracts were lyophilized. These 3 strains were initially assayed against trypsin, chymotrypsin and proteinase K (table 2). In these, *Streptomyces* sp LK3 strain showed high protease inhibitor activity against trypsin and chymotrypsin. The potential strain with 50% inhibiton were further subjected to determine an IC_50_ value. The LK3 extract inhibited trypsin and chymotrypsin with IC_50_ values of 96.77 µg/ml and 161.8 µg/ml respectively (Fig. 2 A).

**Table 2:** Protease inhibitor activity of marine actinobacteria.

**LK1**	90	89	30
**LK2**	91	91	42
**LK3**	98	95	40

Out of 100, 3 isolates exhibited moderate to high protease inhibitor activity of trypsin and chymotrypsin. Many reports are available on the production of inhibitors from *Streptomyces* sp viz., *S.mauvecolor*, *S.michiganensis*, *S.violaceus* and *S. yokosukaensis* were produced antipain against papain enzyme. It is also capable to inhibit trypsin and cathepsin B. *S.hygroscopicus* and *S.lavendulae* produced chymostatin against chymotrypsin. *S.griseoruber* produced elastatinal against elastase [27], [28]. So far, only few reports only available for anti- Pf activity of marine actinobacteria. Maskey *et al*. (2004) reported that the trioxacarcins A and D isolated from the marine *Streptomyces* sp. isolate B8652 BCC 5149 possessed “extremely high antiplasmodial activity” against the parasite *P. falciparum* K1 and NF54 strains which was much higher than the clinically used compound chloroquine [29]. Peraud (2006) reported that the manzamines isolated from the marine *Micromonospora* sp. strain M42 possessed antimalarial activity [30]. Prudhomme *et al* (2008) reported that the salinosporamide A, isolated from the marine *Salinispora tropica* possessed antimalarial activity [31]. It is also acts as a potent proteasome inhibitor in development for treating cancer [32].

### Mode of protease inhibition

The mode of inhibition of peptide on trypsin activity was analyzed using LB plot. The double-reciprocal plot displayed non-competitive inhibition mode (Fig. 2B) as there is a continous steep decline in Vmax with an increase in inhibitor concentration with K_m_ value remaining confined (Table 3). Enzyme activity of trypsin was completely recovered after the dialysis, as showed by the enzyme mixed inhibitor curve (EID) which was similar to the curves of enzyme control without dialysis (EC) and dialysis (ED) (Fig. 2C). A dialyzed mixture of enzyme and extract confirmed that processes are not affected the enzyme activity dialysis. However, the non-dialyzed mixture of enzyme and extract (EIC) showed less inhibited activity and it confirms the irreversible nature of protease inhibitor.

**Table 3:** Kinetic analysis of protease inhibition by peptide.

Peptide (mg/ml)	V_max_ (mM/min)	Km (mM)
**0**	0.24	**1.64**
**0.5**	0.19	**1.54**
**1**	0.14	**1.65**
**2**	0.10	**1.7**

The main role of irreversible inhibitor is once target enzyme inhibited, it cannot reactivate and the organism must reproduce the enzyme. Several reversible protease inhibitors reached the market as a drug but only few drugs of irreversible protease inhibitors are available such as aspirin and β-lactam antibiotics. Sajid and McKerrow (2002) reported irreversible inhibitor could be used in bacterial, viral and parasitic diseases and in the future, irreversible inhibitors are promising source for disease treatment [33].

### Identification of potential strain

**Figure 3:**
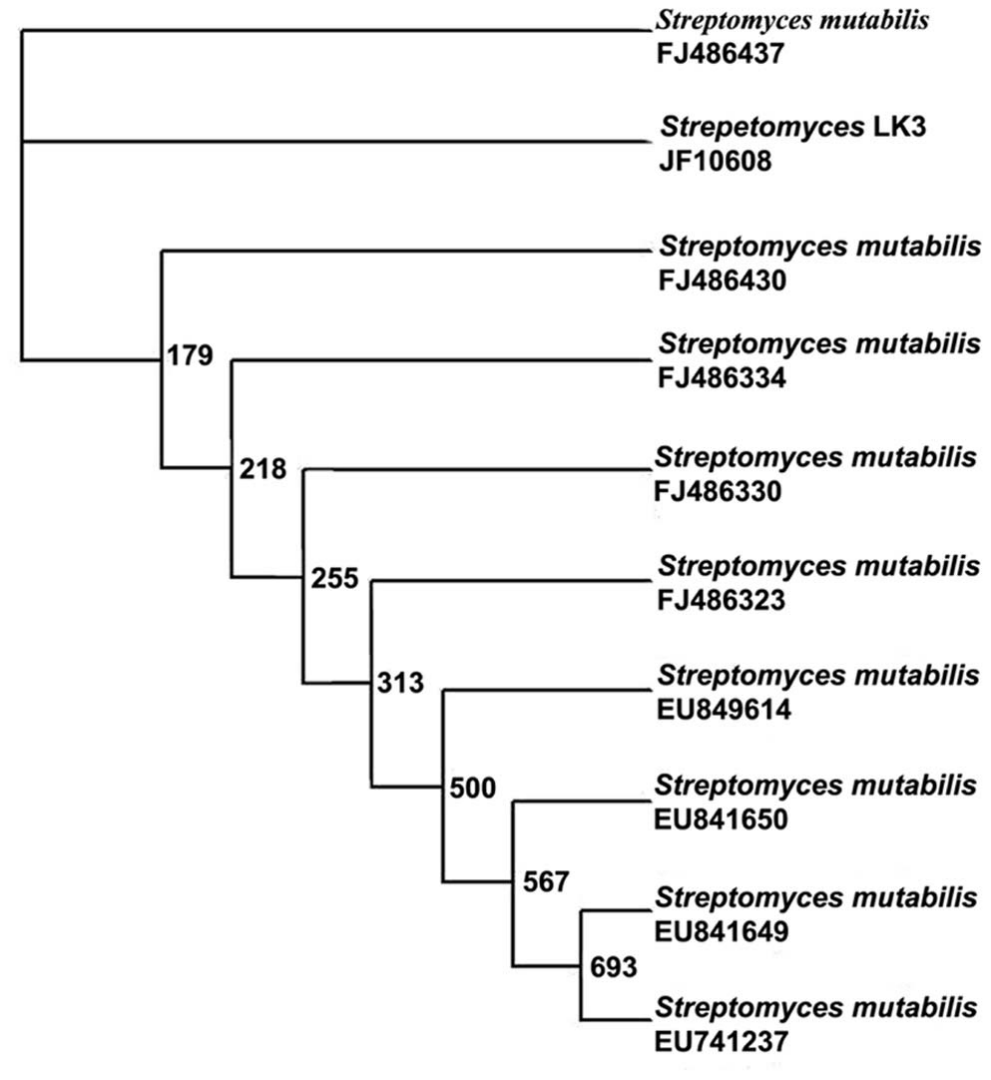
The 16s RNA sequencing confirmed the strain LK3 is a novel species with G+C content of the isolated DNA at 57.29 mol%.

The 16s rRNA sequencing is a method of choice for tracing bacterial phylogeny and definite the taxonomy [34]. Hence, the potential strain was identified as *Streptomyces* sp LK-3 through 16S rRNA Sequencing. The BLAST search showed only 93% similarity with *Streptomyces mutabilis*. The strain was deposited in the Bankit (GenBank, NCBI, USA) under the accession number JF710608. The 16s rRNA sequencing of strain LK3 was confirmed that it occupies a distinctive phylogenetic position within the radiation including representatives of the family Streptomycetes using neighbor-joining tree (Fig. 3) and it forms a separate subclade from other member of *Streptomyces*. It confirms the strain LK3 is a novel species. The G+C content of the isolated DNA is 57.29 mol% (http://tubic.tju.edu.cn/GC-Profile/). Based on the chemotaxonomy, morphological and 16S rRNA gene sequence data suggest that strain LK3 represents a novel species when compared with type strains of species in nonomura key. The polyphasic taxonomic study of strain LK3 merits classification as the type strain of a novel species within the genus *Streptomyces*, and hence the strain were named as *Streptomyces* sp LK3.

### Purification and characterization of protease inhibitor

The RSM optimized *Streptomyces* sp LK3 [35] was subjected to fermentation (7 days) and extracted protease inhibitor was purified by a four sub-sequential steps viz., Ammonium sulphate precipitation, dialysis, Ion exchange chromatography and preparative HPLC chromatography. The protease inhibitor was achieved 80% saturation. Following this, dialysis and anion exchange chromatography was performed.

**Figure 4:**
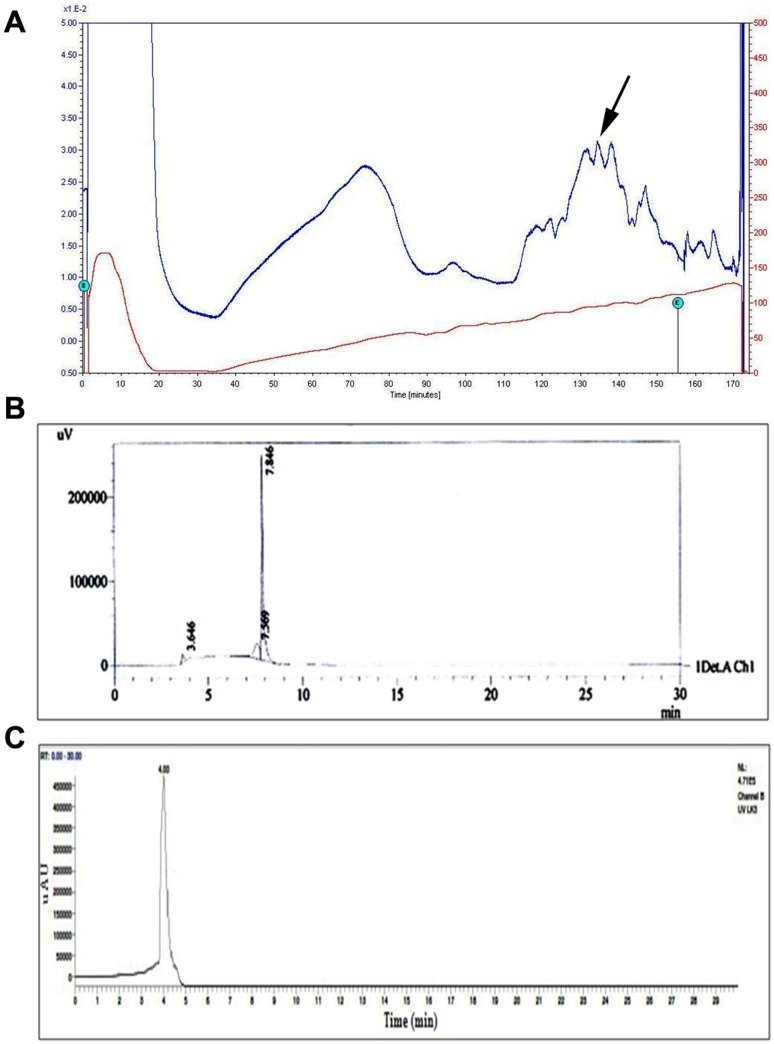
Purification of protease inhibitor. **A.** Elution pattern of protease inhibitor on DEAE-Sepharose anion exchange column; **B**. Elution pattern of protease inhibitor on C18-RP HPLC column; **C**. LCMS analysis of purified compound

The eighth peak contains the highest protein concentration (0.15 mg/ml). Similarly, Angelova *et al* (2005) was purified and characterized a novel protease inhibitor (PISC-2002) isolated from culture supernatants of *Streptomyces chromofuscus* using DEAE-Sepharose [36]. Figure 4A shows the elution pattern of the protease inhibitor and a broad active peak was obtained, when the dialyzed sample was passed through a DEAE Sepharose column with 0.4 M NaCl Tris-HCl buffer with NaCl pH 7.5. Further purification was carried out by preparative HPLC on a C_18_ column (Fig. 4B) and a single peak was confirmed by LCMS (Fig. 4C). About 10 mg of pure protease inhibitor was obtained from 3 g of lyophilised supernatant.

**Figure 5:**
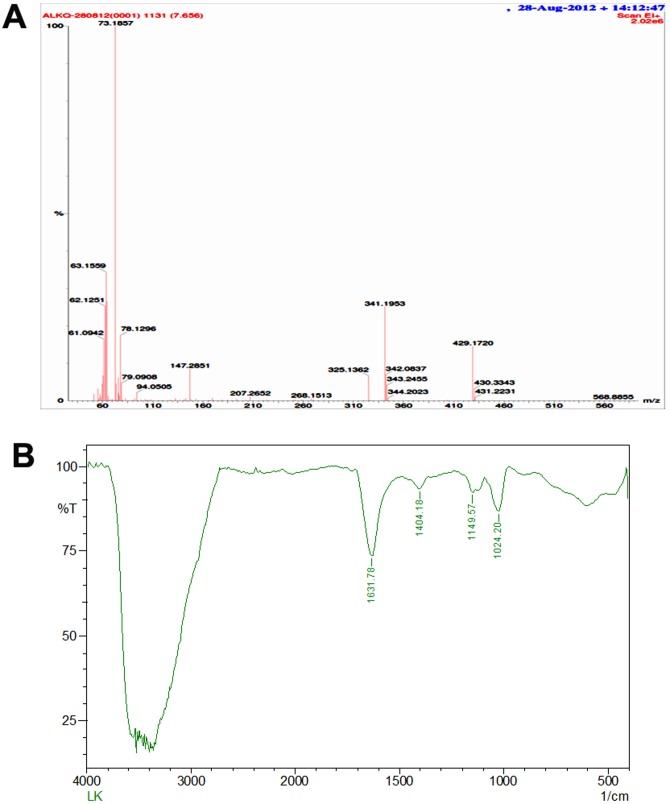
Characterization of protease inhibitor. **A.** Mass spectrum of peptide compound obtained from the GCMS clearly revealed that it may be a small peptide with 5 or 6 aminoacids (Molecular Weight-568da) The amino acids may be present in the peptide are: AILKRVMGNC; **B.** FTIR spectrum of peptide compound. Absorbance at 1632 corresponded to the carbonyl functions whereas the absorbance from 3300–3600cm-1 may be due to the NH function from the aminoacid functional groups.

Mass spectrum obtained from the GCMS clearly revealed that it may be a small peptide with 5 or 6 Aminoacids (Molecular Weight-568da) (Fig. 5A). The ^1^H, ^13^C, H,H-COSY, HSQC also showed the chemical shift values in the aliphatic region (2.5–4.0 and 5.0). This chemical shift may be due to the alpha hydrogens and NH hydrogens. The compound doesn't have any chemical shift values in the aromatic region. The careful examination of IR spectra also revealed the presence of peptides. The IR spectra showed absorbance in the region of 1632 cm^−1^, 3300–3600 cm^−1^ (Fig. 5B). The absorbance around the 1632 corresponded to the carbonyl functions where as the absorbance between 3300–3600 cm^−1^ may be due to the NH function. Apart from this two characteristic peak, no other characteristic peaks have been observed. The amino acids may be present in the peptide are: AILKRVMGNC.

Many reports are available on the production of inhibitors from *Streptomyces* sp viz., *S.mauvecolor*, *S.michiganensis*, *S.violaceus* and *S.yokosukaensis* were produced antipain against papain enzyme and it can also capable to inhibit trypsin and cathepsin B [37], [38]. Inhibitors from *Streptomyces* sp was classified as SSI family inhibitors and designated as SSI –like [39]. Hence, the protease inhibitor from *Streptomyces* sp LK3 was belongs to the SSI family inhibitors.

The microbial origin inhibitors are low molecular weight peptides of unusual structures [40]. Similarly, protease inhibitor from *Streptomyces* sp LK3 is a low molecular weight peptide of unusual structure. In 1969, Umezawa discovered first low molecular weight enzyme inhibitor from *Streptomyces* sp [41]. At present, more than 100 inhibitors were reported but this is the first report on marine *Streptomyces* sp.

### *In vitro* anti- Pf activity

A total of 3 different actinobacterial isolates were chosen for anti- Pf activity based on the protease inhibitor activity. Extracts were tested at five initial concentrations: 20 µg/ml, 40 µg/ml, 60 µg/ml, 80 µg/ml and 100 µg/ml. Three extracts showed a 50 to 85% inhibition. The peptide from *Streptomyces* sp LK3 extract showed significant anti plasmodial activity (IC_50_: 25.78 µg/ml). The rest of the other actinobacterial extracts showed weak activity (IC_50_ >200 µg/ml).

### *In vivo* anti- Pf activity

#### Effect of LK1, LK2 and LK3 on survival and parasite clearance efficacy during *Plasmodium berghei* ANKA infection

**Figure 6:**
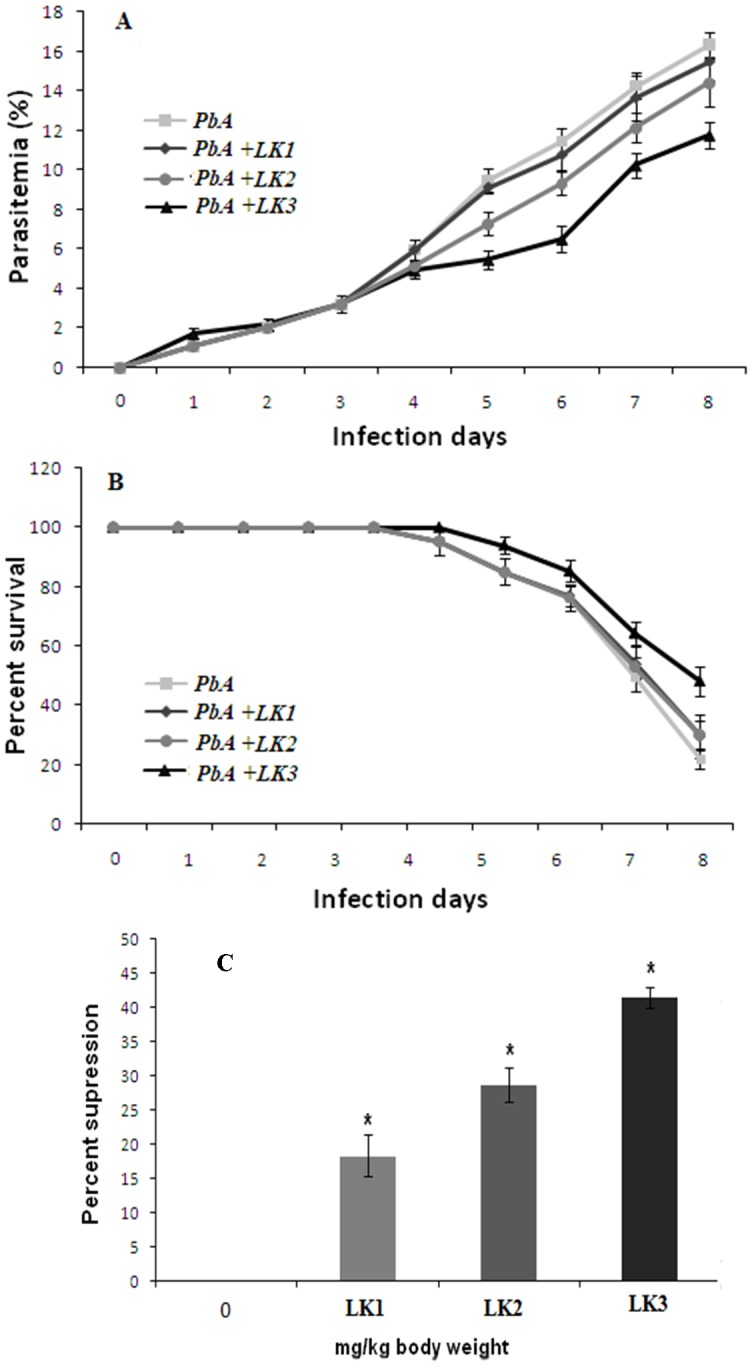
Characteristics of *PbA* infection in Swiss Albino Mice and effect of LK1 (800 mg/kg/BW), LK2 (1000 mg/kg/BW) and LK3 (600 mg/kg/BW) respectively. (A) Resulting parasitemias were expressed as a percent parasitemia (mean ± SD), measured daily on Giemsa stained blood smears. Parasitemia was evaluated statistically by Two-way ANOVA with Bonferroni post-test showed that the parasitemias were significantly different (*p<0.05) between *PbA* infected groups and LK3 treated groups, whereas parasitemias were not significantly different in the LK1, LK-2 group w.r.t. *PbA* infected controls and the matched controls (P>0.05). (B) Effect of LK1 (800 mg/kg/BW), LK2 (1000 mg/kg/BW) and LK3 (600 mg/kg/BW) respectively on survival of mice infected with *P. berghei ANKA*. Each value in Y axis represents the percentage (%) of survival of the treated groups compared to *PbA* treated control mice. The treatment groups represent mice injected intraperitoneally from 2 dpi till the last day of survival. Results are presented as arithmetic mean (±SE) of five mice per group. (C) Percentage parasitemia suppression after administration of LK1 (800 mg/Kg), LK2 (1000 mg/Kg) and LK3 (600 mg/Kg) respectively. Data from five replicates are presented as arithmetic mean ± standard deviation and indicate p<0.05 with respect to the controls. Data shown are one representative experiment of five (n).

The clinical course of infection in the experimental mice is summarized in Figure 5. Mice from both experimental groups were age matched and had, *PbA* infected + LK2 treated set and in *PbA* infected + LK3 treated set (Fig. 6A) and continued to rise till mice dies. Most of the mice succumbed to CM during 4–9 dpi, except in LK3 treated group (*p<0.05) compared to other experimental groups and matched controls. Statistically significant differences in the percentage survival of mice were observed (Fig. 6B).

The ‘8 -day suppressive test’ were adopted for the determination of the percentage of parasite clearance. The doses were LK1 (800 mg/kg/BW), LK2 (1000 mg/kg/BW) and LK3 (600 mg/kg/BW) respectively. Groups of 5 mice each were administered vehicle only. The results of the study indicated that *in vivo*, LK3 displayed a considerable activity against the *P. berghei* ANKA malaria parasite (Fig. 6C).

#### *PbA* infection induces histological changes in the spleen

**Figure 7:**
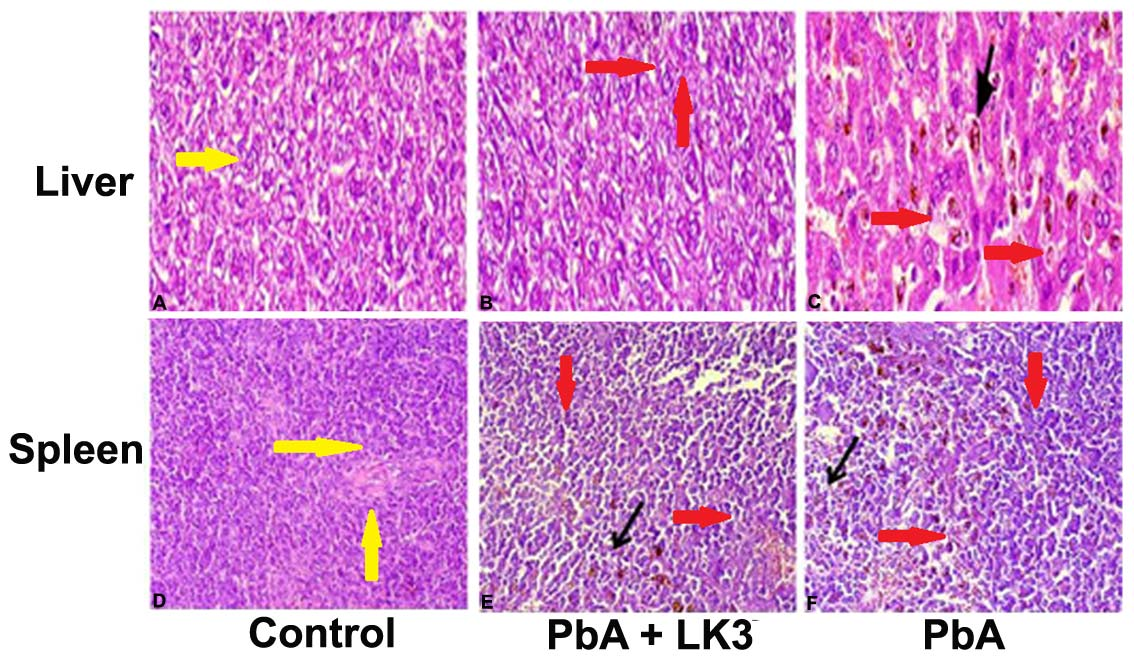
Histopathological changes in hepatic and splenic tissues in response to LK3 treatment during *PbA* infection. Hematoxylin and eosin stains were used to prepare sections of the liver and spleen from mice treated with vehicle, *PbA* infected + 600 mg/Kg LK3 and *PbA* infected respectively. Sections of control (A) and *PbA*+LK3 infected (B) dpi liver showing, surrounding hepatocytes with nuclei and blood sinusoids lined with Kupffer cells. Liver from infected mice (C) shows, moriform vacuolization of hepatocytes and hemozoin pigmented Kupffer cells on 8 dpi. In control (D) and *PbA*+ LK3 infected spleen tissue section (E), distinct and defined separate cluster of white pulp marked by yellow arrows. In treatment tissue sections white pulp and red pulp are not distinct (F). Appearances of hollow spaces without cells observed in spleen tissue sections compared to that of control (red arrows). Black arrows represent hemozoin accumulation in liver and spleen tissue sections. Magnification indicated 40×

To confirm the unpleasant effect of *PbA* infection and effect of LK3 on the spleen and liver, mice were sacrificed, spleen and liver tissues were isolated on 8 dpi, along with controls and HE staining was done. Liver sections of uninfected mice (Fig. 7 A) showed typical architecture and color, clean sinusoids with few cells and resting Kupffer cells. On day 8 of infection, *PbA* infected liver sections showed extensive histopathological changes, presented an enlarged liver laden with malaria pigment (Fig. 7C). In contrast, in liver sections of LK3 treated mice shows significant reduction of malarial pigment and the mice were able to survive the infection (Fig. 7B). Spleen of control mice presented distinct T and B cell zones in the white pulp, resting B-cell follicles, with small lymphocytes, surrounded by well defined marginal zones (MZ), trabeculae (T) and red pulp (RP)(Fig. 7D). In the spleens of *PbA* infected hosts, cells in the white pulp had proliferated considerably and enlarged to the limits wherein the margin between white and red pulp began to disappear and hollow spaces without cells appeared. Follicle germinal centers (Gc) lost the typical architecture acquiring a disorganized aspect, and several phagocytic-centers with hemozoin (Hz) accumulation (Fig.7 F). In *PbA* infected + LK3 treated set, we observed less damaged white pulp and red pulp, small clusters of hemozoin pigments compared to *PbA* infected and control mice (Fig. 7E).

**Figure 8:**
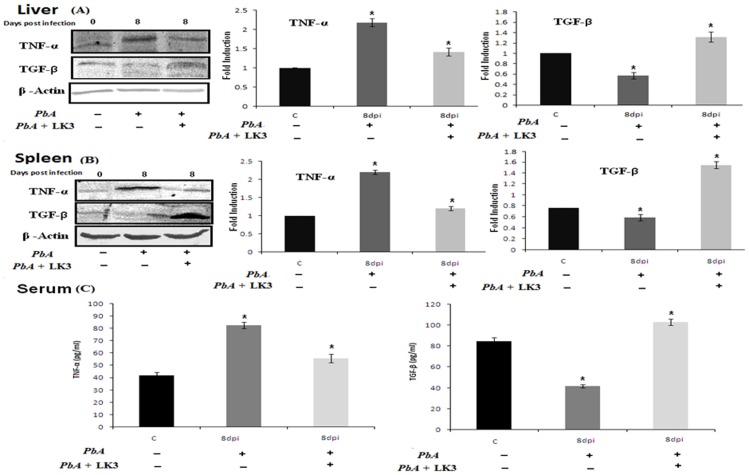
Cell lysate from respective control, treatment spleen and liver were subjected to western blot analysis and ELISA (A) liver and (B) spleen during *PbA* infection and treated with peptide (C) ELISA results (p<0.05, ANOVA followed by post hoc LSD test).

TGF-β a known anti-inflammatory agent, acts to down regulate the production of proinflamatory cytokines. On the other hand much of the pathology of malaria is mediated by proinflamatory cytokine such as TNF-α. From western blot results, significant upregulation of TNF-α and down regulation of TGF-β were observed in the spleen and liver during *PbA* infection with respect to control. The ELISA results were strongly supported by statistically significant western blot results. These observations further support the proposed hypothesis, suggesting an antagonist relationship between TNF-α and TGF-β and their involvement during *PbA* infection (Fig. 8).

More recently, artemisinins resistance *P. falciparum* was confirmed at the Cambodia–Thailand border [42], [43]. Hence, there is a need of new drugs. The *in silico* study revealed that protease inhibitors from five actinobacteria liable to show very high affinity towards the respective receptors. Several researchers demonstrated peptidyl fluoromethyl ketone [44], vinyl sulfone [45] and aldehyde inhibitors [46] capable to protect Plasmodium-infected mice against lethal malaria, which act as cysteine protease inhibitors. Likewise, Gelhaus *et al*. (2005) reported Biotinylated dibenzyl aziridine-2,3-dicarboxylate, it can block host erythrocyte rupture and subsequent merozoite release [47]. In 2006, Ersmark *et al* found aspartic proteases also very good antimalarial drug target [48]. More recently, Yeoh *et al* (2007) reported serine protease primes the malaria parasite for red blood cell invasion [49]. Senior (2005) reported HIV aspartic protease inhibitors showed activity against *Plasmodium falciparum* [50]. Protease inhibitors have also drug of choice shown utility against other infectious diseases caused by protozoans. Similarly, protease inhibitor from *Streptomyces* sp LK3 may be the drug of choice against *Plasmodium falciparum*.

**Figure 9:**
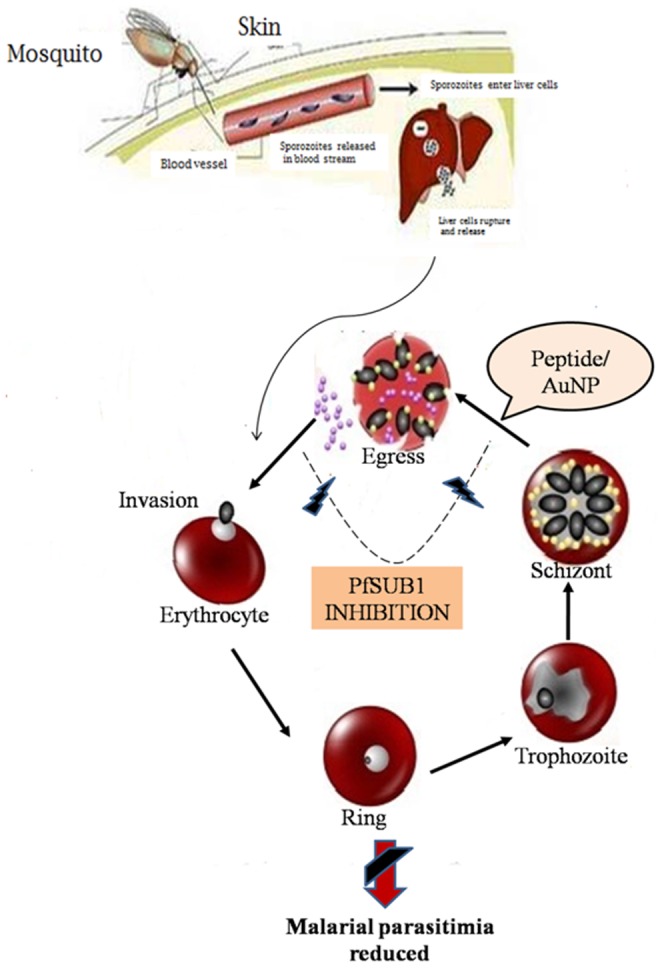
Possible mechanism of peptide from *Streptomyces* sp LK3 against malaria.

McCoubrie et al (2007) and Putrianti et al (2010) reported SERA family members play a major role in malaria life cycle [51], [52]. PfSUB1 is a serine protease involved in both schizont rupture and erythrocyte reinvasion in the *P.falciparum* life cycle. It can be blocked by serine protease inhibitors. The inhibition of PfSUB confirms the egress blocking and reduces the invasion of merozoite. Hence, PfSUB1 is a drug target enzyme [49]. While, compared to other malarial drug target, PfSUB1 is the best choice because no human enzyme homolog is available. Recently, Withers-Martinez et al (2012) also suggested that SUB1 inhibitors could be used in the current dwindling armory of antimalarial agents [53]. Based on our results, we confirmed the protease inhibitor from *Streptomyces* sp LK3 capable of degrading serine protease (Fig. 9).

As anticipated in the *PbA* infected mice in our study along with haemozoin accumulation, spleen histology showed an increase in the cellularity of both red and white pulp and the progressive loss of a defined marginal zone. These expected histological changes were associated with increasing parasite burden. The increase in red pulp cellularity is reported to occur as the phagocytic and erythropoietic activity of the spleen is enhanced to replace infected erythrocytes with a healthy population of cells. Although primate models provide a better prediction of efficacy in human than the rodent models, the latter has also been validated through the identification of several conventional antimalarials, such as chloroquine, halofantrine, mefloquine and more recently artemisinin derivatives. *P. berghei* are used in the prediction of treatment outcomes. Hence, it was an appropriate parasite for the study.

The 8-day suppressive test is a standard test commonly used for antimalarial screening, and the determination of percent inhibition of parasitemia is the most reliable parameter. Therefore, it is clear from the result that in *P. berghei* infected mice treated with the LK1, LK2 and LK3, the percentage of parasitemia changed significantly from those in the control animals in LK3 treated group. This significant suppression of parasitemia by the LK3 group on day 8 was also in agreement with *P. falciparum* isolates NZR-2 *in vitro*. The observed anti- Pf activity is consistent with the traditional use of artemisinin. From the present study, it can be concluded that the LK3 have shown parasite suppressive effects on *P.berghei* infected Swiss albino mice in a dose related fashion, whereas the underlying mechanisms of protease inhibitor LK3 need to study in detail for future therapeutic approach.

However, the studies offer additional justification for the use of protease inhibitors as antimalarials and suggest that differences between *P. falciparum* and *P berghei* targets may contribute to the limited *in vivo* efficacies of some protease inhibitors. The results further suggest a need for rethinking traditional approaches. Anti-malarial drug discovery has typically relied on validation with rodent models before advancement to full development. However, in cases where human and rodent parasite targets differ, it may be appropriate to bring promising compounds forward without validation in rodents, perhaps after extensive pharmacokinetic studies, and then to evaluate their efficacy in *P. falciparum* models.

## Conclusion

The actinobacteria isolate, *Streptomyces* sp LK3 was isolated from Nicobar marine sediment samples, which yielded peptide compound. Peptide exhibits anti- Pf activity in both *in vitro* and *in vivo* experiments. Our results confirmed up-regulation of TGF-β and down-regulation of TNF-α in tissue and serum level in *PbA* infected peptide treated mice compared to *PbA* infection. In conclusion, the peptide is effective in delaying parasitemia rise, survivility caused by *P. berghei* ANKA. The results obtained suggest that the peptide possess anti-Pf activity and could be considered as a potential source for anti-Pf drug development.
